# A new solvate of afatinib, a specific inhibitor of the ErbB family of tyrosine kinases

**DOI:** 10.1107/S2056989017002626

**Published:** 2017-02-21

**Authors:** Matthias Zeller, Gabriel Lima Barros de Araujo, Trev Parker, Amrinder Singh Rai, Stephen R. Byrn

**Affiliations:** a Department of Chemistry, Purdue University, 560 Oval Dr., W. Lafayette, IN 47907-2084, USA; b Faculty of Pharmaceutical Sciences, Department of Pharmacy, University of Sao Paulo, Sao Paulo, SP, Brazil; c Department of Industrial and Physical Pharmacy, Purdue University, West Lafayette, Indiana, USA

**Keywords:** crystal structure, pseudo-symmetry, pseudo-inversion center, ErbB tyrosine kinase inhibitor, hydrogen bonding

## Abstract

Afatinib (systematic name: *N*-{4-(3-chloro-4-fluoro­anilino)-7-[(tetra­hydro­furan-3-yl)­oxy]quinazolin-6-yl}-4-(di­methyl­amino)­but-2-enamide), is a specific in­hibitor of the ErbB family of tyrosine kinases. The free base form crystallizes from aceto­nitrile as a mixed water–aceto­nitrile solvent, C_24_H_25_ClFN_5_O_3_·0.25C_2_H_3_N·2H_2_O. It crystallizes with two independent mol­ecules (*A* and *B*) in the asymmetric unit of the chiral space group *P*42_1_2, but exhibits close to perfect pseudo-inversion symmetry, emulating *P*4/*ncc* that relates the two mol­ecules to each other. Exact inversion symmetry is however broken by swapping of oxygen and CH_2_ moieties of the outer tetra­hydro­furanyl substituents of the two independent mol­ecules. This can, in turn, be traced back to C—H⋯N and C—H⋯O inter­actions of the aceto­nitrile solvent mol­ecules with the tetra­hydro­furan oxygen and CH_2_ units. In the crystal, neighboring mol­ecules are connected *via* N—H⋯O hydrogen bonds between the secondary amine and the amide keto O atom. Additional hydrogen bonds are formed through the water solvent mol­ecules, which are engaged in O—H⋯O and O—H⋯N hydrogen bonds connecting to the di­methyl­amino N atom, the amide keto O atom, and one of the quinazoline N atoms of a neighboring mol­ecule, leading to an intricate three-dimensional hydrogen-bonded superstructure. There are two types of channels stretching along the direction of the *c* axis; one along the fourfold rotational axis, occupied by aceto­nitrile solvent mol­ecules situated on that axis, and parallel channels which are not occupied by any solvent.

## Abstract 2

The water/aceto­nitrile solvent of afatinib exhibits pseudo-inversion symmetry. Exact inversion symmetry is broken by swapping of oxygen and CH_2_ moieties of its tetra­hydro­furanyl substituents, which can be traced back to C—H⋯N and C—H⋯O inter­actions of the aceto­nitrile solvent mol­ecules with the tetra­hydro­furanyl units.

## Chemical context   

Afatinib is an orally administered anti­tumor drug used for the treatment of patients with metastatic nonsmall cell lung carcinoma (Keating, 2014 ▸). This drug is an irreversible specific inhibitor of ErbB family of tyrosine kinases, comprising EGFR (ErbB1), HER2 (ErbB2), and HER4 (ErbB4) (Hirsh, 2011 ▸; Keating, 2014 ▸). It is marketed as a dimaleate salt (Giotrif, Boehringer–Ingelheim Pharma GmbH, Ingelheim, Germany) and is reported to present polymorphism as a free base and in its salt forms, as well as an ethanol solvate (Gidwani *et al.*, 2012 ▸; Jiadeng, 2016 ▸). However, to the best of our knowledge no single-crystal structure has been described so far for any of the reported crystal forms. Herein, we describe an aceto­nitrile–water solvent structure of afatinib free base obtained *via* vapor diffusion experiments.

## Structural commentary   

**Figure 1:**
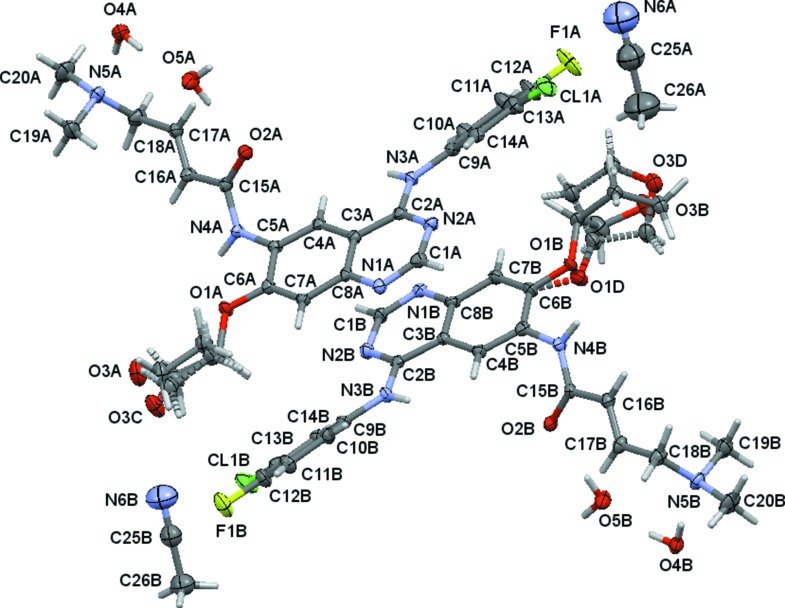
The mol­ecular structure of the title compound, showing the atom labeling. Displacement ellipsoids are drawn at the 50% probability level. Dashed lines indicate minor disordered moieties *C* and *D* of the tetra­hydro­furan-3-yl­oxy units. C-atom labels of the disordered moieties have been omitted for clarity.

**Figure 2:**
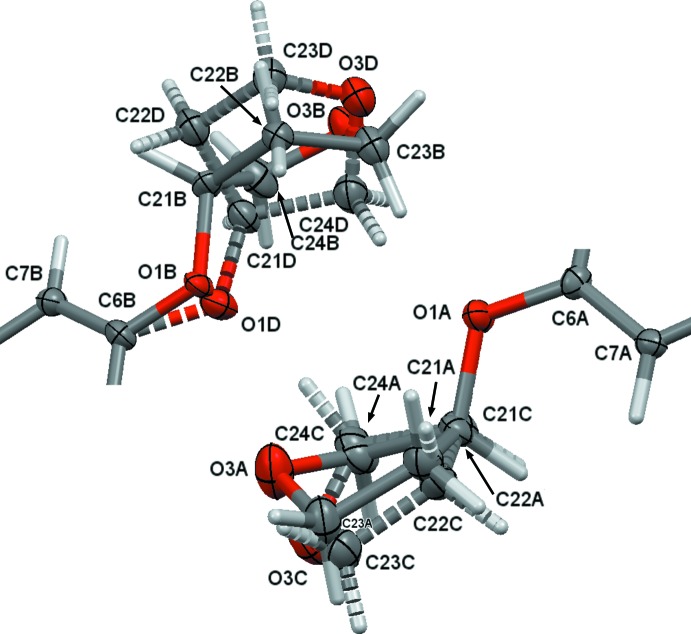
View of the two disordered tetra­hydro­furan-3-yl­oxy moieties, with 50% probability displacement ellipsoids. Dashed lines indicate minor disordered moieties *C* and *D*.

Crystals of free base afatinib were obtained from an aceto­nitrile solution through vapor diffusion of hexa­nes. The compound crystallized in a tetra­gonal setting, space group *P*42_1_2, as a mixed water–aceto­nitrile solvent with two mol­ecules of water and one-quarter mol­ecule of aceto­nitrile per formula unit of afatinib (Fig. 1 ▸). Two crystallographically independent mol­ecules (*A* and *B*) of afatinib are present, and both exhibit disorder of its tetra­hydro­furan-3-yl­oxy units, with a disorder ratio of 0.718 (9):0.282 (9) for mol­ecule *A* and 0.787 (5):0.213 (5) for mol­ecule *B*. The type of disorder differs slightly between the two mol­ecules (see Fig. 2 ▸ and §5, *Refinement*).
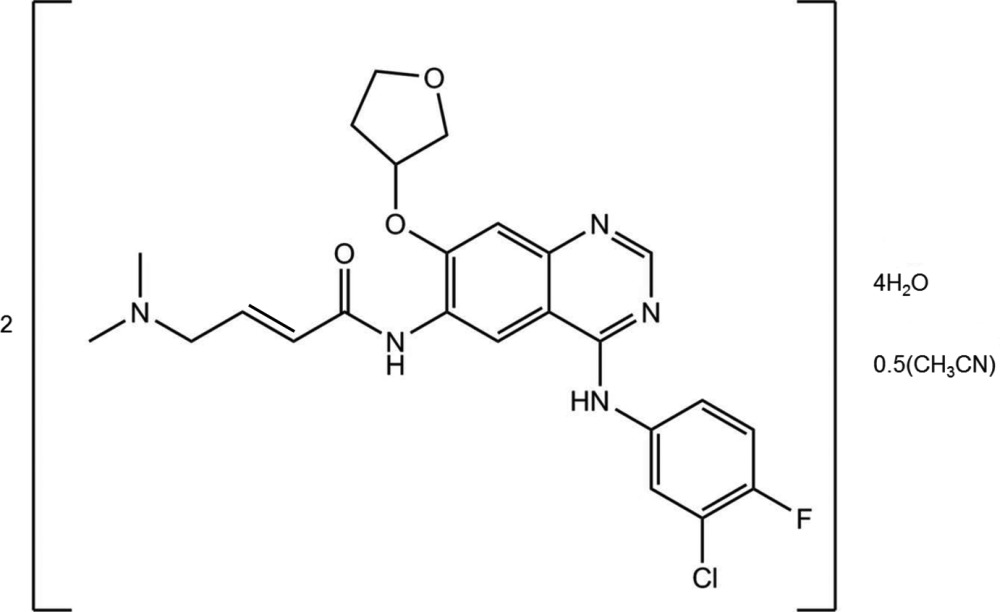


**Figure 3:**
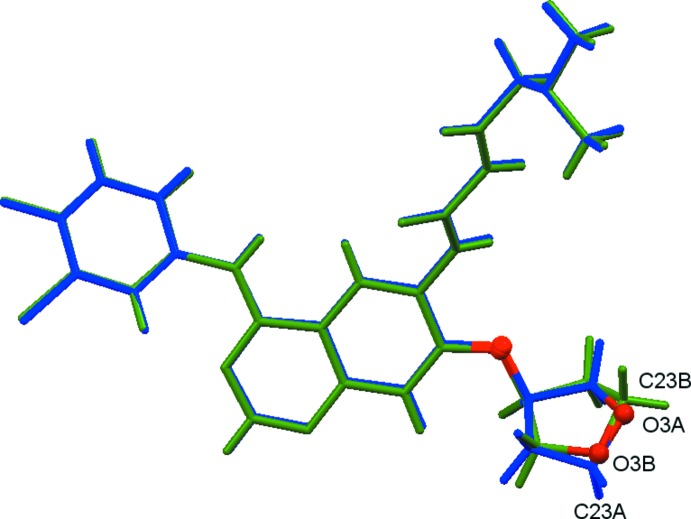
Least-squares overlay of mol­ecule *A* (blue) on inverted mol­ecule *B* (green). O atoms of the tetra­hydro­furan-3-yl­oxy units are shown as red spheres to illustrate the absence of inversion symmetry in the title structure. The r.m.s. deviation is 0.6892 Å.

**Figure 4:**
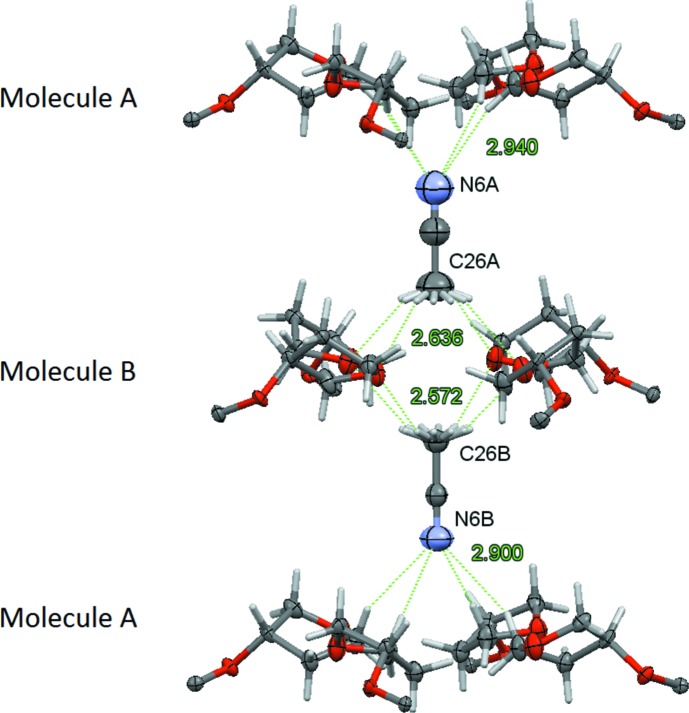
View of the inter­actions of the aceto­nitrile solvent mol­ecules with the tetra­hydro­furanyl units of mol­ecules *A* and *B*. The remaining sections of mol­ecules *A* and *B* have been omitted for clarity. Aceto­nitrile mol­ecules are located on a fourfold rotation axis hence and the H atoms are fourfold disordered. C—H⋯N and C—H⋯O hydrogen bonds are indicated as green dashed lines and the H⋯N and H⋯O distances (Å) are given (see Table 1 ▸).

The two independent mol­ecules (*A* and *B*) are related by a pseudo-inversion center with close to centrosymmetric *P*4/*ncc* symmetry. After inversion, the two mol­ecules are nearly superimposable with only very minor deviations for the aromatic core, the chloro­fluoro­aniline substituent and the (di­methyl­amino)­but-2-enamide unit (see Fig. 3 ▸ for the mol­ecular overlay). Focusing only on the two major moieties exact inversion symmetry is broken solely by the positions of the tetra­hydro­furan (THF) O atoms, which are swapped with a CH_2_ group between the two mol­ecules. Associated with the different positions of O and CH_2_ moieties, and possibly providing an explanation for this observation, is an ordering of the aceto­nitrile solvent mol­ecules. Aceto­nitrile mol­ecules related by pseudo-inversion do not as expected point in opposite directions, but are co-parallel with each other. The CH_3_CN mol­ecules are located in channels surrounded by the tetra­hydro­furanyl units, and they inter­act with mol­ecules *A* and *B* in opposite ways. The methyl ends of both CH_3_CN mol­ecules form C—H⋯O hydrogen bonds with the O atoms of the major moieties of mol­ecule *B*, capping a tetra­mer of THF units on both sides. The nitro­gen ends of the aceto­nitrile units, on the other hand, act as acceptors of weak C—H⋯N hydrogen bonds (Fig. 4 ▸ and Table 1 ▸). The methyl and nitro­gen ends of the linear mol­ecules are related by the pseudo-inversion operation. The different polarity of the two ends, one an hydrogen-bond donor, the other an hydrogen-bond acceptor, can thus be seen as an immediate cause for the swapping of oxygen and the CH_2_ groups, which are also hydrogen-bond donors and acceptors, so that an attractive rather than repulsive inter­action is maintained. Exact inversion symmetry is also broken by the different disorder patterns for the tetra­hydro­furan-3-yl­oxy units substituents (see §5, *Refinement*, and Fig. 2 for details). The absence of inversion symmetry is further evidenced by the value of the absolute structure parameter for racemic twinning, which refined to 0.02 (1), indicating a chiral or noncentrosymmetric space group incompatible with centrosymmetric *P*4/*ncc* symmetry.

Bond lengths and angles in both mol­ecules are unexceptional and in the expected ranges. The central quinazoline cores of the mol­ecules are nearly planar, with maximum deviations of 0.073 (5) Å for atoms C5*A* and C5*B* in mol­ecules *A* and *B*, respectively. The but-2-enamide units are all-*trans* and also nearly planar (r.m.s. deviations are 0.046 Å for mol­ecule *A* and 0.042 Å for mol­ecule *B*, for all non-H atoms including the directly neighboring quinazoline C atom). Their mean planes are inclined to the quinazoline ring by 47.8 (2) and 47.3 (2)° in mol­ecules *A* and *B*, respectively. The chloro­fluoro­aniline rings are also twisted out of the mean plane of the quinazoline ring by 36.6 (2) and 36.9 (1)° for mol­ecules *A* and *B*, respectively.

The simulated powder pattern of the aceto­nitrile–water solvate reported here does not agree with any of the free base forms of afatinib A–D reported in the literature (Gidwani *et al.*, 2012 ▸).

## Supra­molecular features   

**Figure 5:**
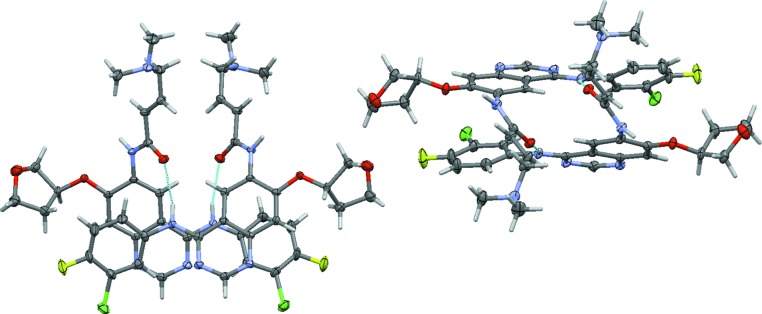
Hydrogen-bonded twofold rotation dimer of *A* mol­ecules, in two oblique views.

**Figure 6:**
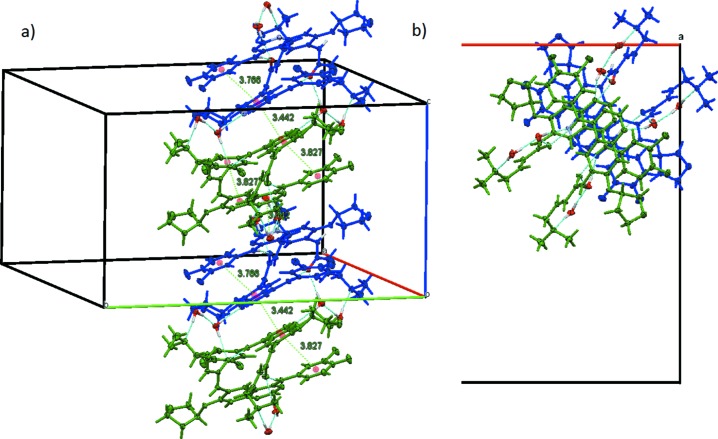
Stacks along the *c*-axis direction connected through hydrogen bonds (blue dashed lines) and π–π stacking inter­actions (green dashed lines). (*a*) Side view with distances (Å) between ring centroids (red spheres) involved in π–π stacking. (*b*) Top view down the *c* axis. Colour code: mol­ecule *A* blue, mol­ecule *B* green.

In the crystal of the title compound, neighboring mol­ecules are connected *via* N—H⋯O hydrogen bonds between the secondary amine and the amide keto O atom (see Table 1 ▸ for details). Mol­ecules are connected through pairwise hydrogen bonds, graph-set motif 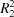
18, to their symmetry-related counterparts, creating twofold rotation symmetric dimers of *A* and *B* mol­ecules, respectively (Fig. 5 ▸). Individual *A*–*A* and *B*–*B* dimers are arranged in infinite stacks along the *c*-axis direction through π-stacking inter­actions between the quinazoline units, and through weaker and more tilted π-stacking inter­actions between the annulated and the fluoro­chloro benzene rings (Fig. 6 ▸). Individual stacking inter­actions between the quinazoline units are across the pseudo-inversion centers, forming close to centrosymmetric pairs of *A*–*B* dimers, with an inter­planar angle between quinazoline units of only 1.24 (11)°. The centroid to centroid distance between the pyrimidine rings of the *A* and *B* mol­ecules is 3.442 (2) Å, the perpendicular distances between the two rings are 3.3144 (18) and 3.3113 (17) Å, with a slippage between the rings of 0.937 Å. The distance between annulated and chloro­fluoro­benzene rings is at 3.827 (3) Å substanti­ally larger, and the slippage is 1.506 Å. The stacks created along the *c*-axis direction are further stabilized *via* hydrogen bonds involving the solvent water mol­ecules. The O5 water mol­ecule hydrogen bonds to the N1 atom of the quinazoline unit, connecting every second mol­ecule in the infinite stacks, and the O4 water mol­ecule hydrogen bonds to the di­methyl­amine N atom and water mol­ecule O5, thus bridging the two ends of the di­methyl­amino­but-2-enamide units, giving the stacks additional support and stiffness (see Table 1 ▸ for details).

**Figure 7:**
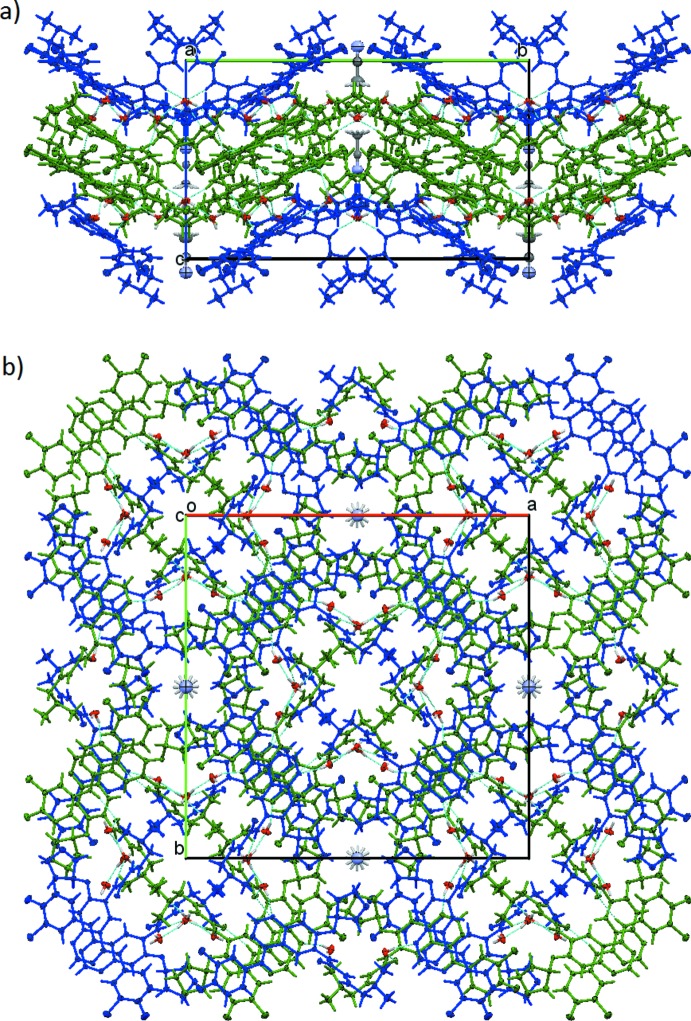
Crystal packing viewed along the *a* axis, showing the inter­digitation of parallel stacks along the *c*-axis direction (see Fig. 4 ▸) and the hydrogen bonds (dashed lines) connecting them, as well as aceto­nitrile occupied and empty channels at the *A*- and *B*-faces and the center of the unit cell, respectively.

The four parallel stacks within each unit cell are inter­digitating with each other, and are also connected through another hydrogen bond facilitated by the water mol­ecule, which acts as a hydrogen-bonding acceptor for the amide N—H group. Additional weaker C—H⋯N and C—H⋯O inter­actions (Table 1 ▸) also contribute to the structure and lattice stability (see Table 1 ▸ for details). In combination, these inter­actions lead to an intricate three-dimensional superstructure facilitated by hydrogen bonding, π-stacking and inter­digitation of mol­ecule side arms (Fig. 7 ▸). In between the connected infinite stacks there remains some void space in the form of channels that stretch along the *c*-axis direction. Two different types of channels are found: channels along the fourfold rotational axis that are occupied by the aceto­nitrile solvent mol­ecules, with the solvent mol­ecules situated on that axis, and another set of parallel channels that stretch directly along the *c*-axis direction and are not occupied by any solvent.

## Synthesis and crystallization   

High-purity afatinib free base (>99%) was acquired from LC Labs and high-purity solvents (aceto­nitrile and hexa­nes) were procured from Sigma–Aldrich (Sigma–Aldrich, St Louis, MO, USA). Crystals suitable for single X-ray diffraction studies were obtained by vapor diffusion (Spingler *et al.*, 2012 ▸), where afatinib free base was solubilized in aceto­nitrile and exposed to vapor of hexa­nes in a closed system.

## Refinement   

Crystal data, data collection and structure refinement details are summarized in Table 2 ▸. The structure exhibits pseudo-inversion symmetry emulating the space group *P*4/*ncc*, with two independent mol­ecules, indicated by label suffixes *A* and *B*, in the asymmetric unit that are nearly related by a pseudo-inversion center. Exact inversion symmetry is not realized, as evidenced by the BASF value for racemic twinning, which refined to 0.02 (1). Exact inversion symmetry is broken by flipping of the THF ring, exchanging O and CH_2_ units, and by a different disorder pattern for the tetra­hydro­furanyl substituents of the two independent mol­ecules.

For the first mol­ecule, suffix *A*, the two disordered moieties differ mostly by the position of the tetra­hydro­furanyl O atom, which forms the flap of the THF’s envelope conformation, and is bent to opposite sides for the two moieties. The ether O atom and the THF C atoms are only slightly shifted between the two disordered moieties. For mol­ecule *B*, the disorder is more pronounced and extends to the ether oxygen atom. The THF ring is mirror imaged between the two disordered units, swapping the positions of the O atom with that of a methyl­ene group and shifting the two units against each other.

All four THF moieties were restrained to have similar geometries (SAME commands of *SHELXL2016*; Sheldrick, 2015 ▸), and *U_ij_* components of the anisotropic displacement parameters were restrained to be similar for disordered atoms closer to each other than 1.7 Å (SIMU commands of *SHELXL2016*; Sheldrick, 2015 ▸). The occupancy ratio refined to 0.718 (9):0.282 (9) for moieties *A* and *C*, and to 0.787 (5):0.213 (5) for moieties *B* and *D*.

The water H atoms were located in difference Fourier maps and refined with a distance restraint of O—H = 0.84 (2) Å, and C- and N-bound H atoms were placed in calculated positions and treated as riding, with C—H = 0.95–0.99 Å and N—H = 0.88 Å, and with *U*_iso_(H) = 1.5*U*_eq_(O,C-meth­yl) and 1.2*U*_eq_(C,N) for other H atoms. Aceto­nitrile mol­ecules are located on fourfold axes and the H atoms are fourfold disordered by symmetry.

## Supplementary Material

Crystal structure: contains datablock(s) I, global. DOI: 10.1107/S2056989017002626/su5351sup1.cif

Structure factors: contains datablock(s) I. DOI: 10.1107/S2056989017002626/su5351Isup2.hkl

CCDC reference: 1532940

Additional supporting information:  crystallographic information; 3D view; checkCIF report

## Figures and Tables

**Table 1:** Hydrogen-bond geometry (Å, °)

*D*—H⋯*A*	*D*—H	H⋯*A*	*D*⋯*A*	*D*—H⋯*A*
N4*A*—H4*A*1⋯O4*B* ^i^	0.88	1.94	2.804 (5)	166
N3*A*—H3*A*⋯O2*A* ^ii^	0.88	2.22	3.088 (5)	167
O4*A*—H4*C*⋯N5*A*	0.85 (5)	1.97 (5)	2.816 (6)	173 (4)
O4*A*—H4*D*⋯O5*A*	0.86 (5)	1.91 (5)	2.759 (6)	169 (5)
O5*A*—H5*C*⋯O2*A*	0.85 (3)	2.00 (4)	2.844 (4)	174 (5)
O5*A*—H5*D*⋯N1*B* ^ii^	0.83 (5)	1.98 (5)	2.775 (6)	161 (5)
C4*A*—H4*A*⋯O2*A* ^ii^	0.95	2.31	3.246 (6)	168
C16*A*—H16*A*⋯O4*B* ^i^	0.95	2.41	3.183 (6)	139
C22*A*—H22*A*⋯O3*A* ^iii^	0.99	2.40	3.353 (13)	162
C24*A*—H24*A*⋯O5*B* ^i^	0.99	2.50	3.388 (14)	150
N4*B*—H4*B*1⋯O4*A* ^iv^	0.88	1.96	2.820 (5)	167
N3*B*—H3*B*⋯O2*B* ^v^	0.88	2.26	3.123 (5)	166
O4*B*—H4*E*⋯N5*B*	0.87 (5)	1.95 (5)	2.811 (5)	175 (6)
O4*B*—H4*F*⋯O5*B*	0.86 (4)	1.91 (4)	2.756 (5)	169 (4)
O5*B*—H5*E*⋯O2*B*	0.85 (4)	1.98 (3)	2.828 (4)	173 (5)
O5*B*—H5*F*⋯N1*A* ^v^	0.86 (4)	1.95 (4)	2.794 (5)	169 (5)
C4*B*—H4*B*⋯O2*B* ^v^	0.95	2.34	3.280 (6)	169
C16*B*—H16*B*⋯O4*A* ^iv^	0.95	2.42	3.197 (6)	139
C22*B*—H22*D*⋯O5*A* ^iv^	0.99	2.43	3.160 (8)	130
C24*B*—H24*D*⋯O3*B* ^vi^	0.99	2.57	3.455 (9)	149

Symmetry codes: (i) 
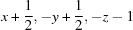
; (ii) 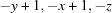; (iii) 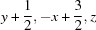; (iv) 
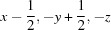
; (v) 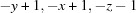; (vi) 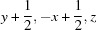.

**Table 2:** Experimental details

Crystal data	
Chemical formula	2C_24_H_25_ClFN_5_O_3_·0.5C_2_H_3_N·4H_2_O
*M*_r_	1064.47
Crystal system, space group	Tetragonal, *P*42_1_2
Temperature (K)	100
*a*, *c* (Å)	26.2427 (4), 15.1639 (3)
*V* (Å^3^)	10443.1 (4)
*Z*	8
Radiation type	Cu *K*α
μ (mm^−1^)	1.75
Crystal size (mm)	0.35 × 0.15 × 0.11

Data collection	
Diffractometer	Rigaku Rapid II curved image plate
Absorption correction	Multi-scan (*SCALEPACK*; Otwinowski & Minor, 1997 ▸)
*T*_min_, *T*_max_	0.681, 0.831
No. of measured, independent and observed [*I* > 2σ(*I*)] reflections	50059, 9958, 7679
*R*_int_	0.056
(sin θ/λ)_max_ (Å^−1^)	0.617

Refinement	
*R*[*F* ^2^ > 2σ(*F* ^2^)], *wR*(*F* ^2^), *S*	0.049, 0.122, 1.03
No. of reflections	9958
No. of parameters	797
No. of restraints	414
H-atom treatment	H atoms treated by a mixture of independent and constrained refinement
Δρ_max_, Δρ_min_ (e Å^−3^)	0.30, −0.40
Absolute structure	Flack *x* determined using 2765 quotients [(*I* ^+^)−(*I* ^−^)]/[(*I* ^+^)+(*I* ^−^)] (Parsons *et al.*, 2013 ▸)
Absolute structure parameter	0.02 (1)

Computer programs: *HKL-3000* (Otwinowski & Minor, 1997 ▸), *SHELXS97* (Sheldrick, 2008 ▸), *SHELXL2016* (Sheldrick, 2015 ▸), *SHELXLE* (Hübschle *et al.*, 2011 ▸), *Mercury* (Macrae *et al.*, 2008 ▸) and *publCIF* (Westrip, 2010 ▸).

## supplementary crystallographic information

### Crystal data

**Table:**

2C_24_H_25_ClFN_5_O_3_·0.5C_2_H_3_N·4H_2_O	*D*_x_ = 1.354 Mg m^−^^3^
*M**_r_* = 1064.47	Cu *K*α radiation, λ = 1.54178 Å
Tetragonal, *P*42_1_2	Cell parameters from 50059 reflections
*a* = 26.2427 (4) Å	θ = 3.4–72.1°
*c* = 15.1639 (3) Å	µ = 1.75 mm^−^^1^
*V* = 10443.1 (4) Å^3^	*T* = 100 K
*Z* = 8	Needle, colorless
*F*(000) = 4472	0.35 × 0.15 × 0.11 mm

### Data collection

**Table:**

Rigaku Rapid II curved image plate diffractometer	9958 independent reflections
Radiation source: microfocus X-ray tube	7679 reflections with *I* > 2σ(*I*)
Laterally graded multilayer (Goebel) mirror monochromator	*R*_int_ = 0.056
ω scans	θ_max_ = 72.1°, θ_min_ = 3.4°
Absorption correction: multi-scan (SCALEPACK; Otwinowski & Minor, 1997)	*h* = −32→29
*T*_min_ = 0.681, *T*_max_ = 0.831	*k* = −26→32
50059 measured reflections	*l* = −17→18

### Refinement

**Table:**

Refinement on *F*^2^	Hydrogen site location: mixed
Least-squares matrix: full	H atoms treated by a mixture of independent and constrained refinement
*R*[*F*^2^ > 2σ(*F*^2^)] = 0.049	*w* = 1/[σ^2^(*F*_o_^2^) + (0.043*P*)^2^ + 7.7055*P*] where *P* = (*F*_o_^2^ + 2*F*_c_^2^)/3
*wR*(*F*^2^) = 0.122	(Δ/σ)_max_ < 0.001
*S* = 1.03	Δρ_max_ = 0.30 e Å^−^^3^
9958 reflections	Δρ_min_ = −0.40 e Å^−^^3^
797 parameters	Extinction correction: (SHELXL2016; Sheldrick, 2015), Fc^*^=kFc[1+0.001xFc^2^λ^3^/sin(2θ)]^-1/4^
414 restraints	Extinction coefficient: 0.00033 (4)
Primary atom site location: structure-invariant direct methods	Absolute structure: Flack *x* determined using 2765 quotients [(I+)-(I-)]/[(I+)+(I-)] (Parsons *et al.*, 2013)
Secondary atom site location: difference Fourier map	Absolute structure parameter: 0.02 (1)

### Special details

**Table:**

Geometry. All esds (except the esd in the dihedral angle between two l.s. planes) are estimated using the full covariance matrix. The cell esds are taken into account individually in the estimation of esds in distances, angles and torsion angles; correlations between esds in cell parameters are only used when they are defined by crystal symmetry. An approximate (isotropic) treatment of cell esds is used for estimating esds involving l.s. planes.
Refinement. The structure exhibits pseudo-inversion symmetry emulating the space group P4/ncc. Exact inversion symmetry is not realized (BASF value for racemic twinning 0.020 (10)). Two tetrahydrofuran rings (related by pseudo-inversion) are disordered in differing ways. For one the ring is inverted at the oxygen. For the other the ring is mirror imaged swapping the position of the oxygen atom. All four moieties were restrained to have similar geometries, and Uij components of ADPs were restrained to be similar for disordered atoms closer to each other than 1.7 Angstrom. Occupancy ratios refined to 0.718 (9) to 0.282 (9) for moieties A and C, and to 0.787 (5) to 0.213 (5) for moieties B and D. Water H atom O-H bond lengths were restrained to 0.84 (2) Angstrom.

### Fractional atomic coordinates and isotropic or equivalent isotropic displacement parameters (Å^2^)

**Table:**

	*x*	*y*	*z*	*U*_iso_*/*U*_eq_	Occ. (<1)
Cl1A	0.54599 (6)	0.23028 (6)	0.07047 (8)	0.0360 (4)	
F1A	0.53997 (14)	0.11926 (14)	0.0581 (2)	0.0539 (11)	
N1A	0.75215 (15)	0.32939 (16)	−0.2041 (2)	0.0181 (9)	
N2A	0.69450 (16)	0.27266 (17)	−0.1303 (2)	0.0203 (10)	
N3A	0.72103 (16)	0.19492 (16)	−0.0728 (2)	0.0184 (10)	
H3A	0.746818	0.173820	−0.066170	0.022*	
C1A	0.7073 (2)	0.3151 (2)	−0.1742 (3)	0.0201 (12)	
H1A	0.680162	0.338100	−0.185363	0.024*	
C2A	0.7321 (2)	0.24006 (19)	−0.1137 (3)	0.0168 (11)	
C3A	0.78385 (19)	0.25115 (19)	−0.1379 (3)	0.0170 (12)	
C4A	0.82582 (19)	0.22136 (19)	−0.1152 (3)	0.0153 (11)	
H4A	0.820572	0.190478	−0.083829	0.018*	
N4A	0.91692 (16)	0.20715 (16)	−0.1080 (2)	0.0166 (10)	
H4A1	0.941274	0.200066	−0.145975	0.020*	
C5A	0.87443 (19)	0.2355 (2)	−0.1371 (3)	0.0165 (11)	
N5A	1.04992 (17)	0.12854 (18)	0.1606 (3)	0.0254 (11)	
C6A	0.88205 (19)	0.2798 (2)	−0.1896 (3)	0.0166 (11)	
C7A	0.84157 (19)	0.31025 (19)	−0.2122 (3)	0.0170 (11)	
H7A	0.846941	0.340077	−0.246439	0.020*	
C8A	0.79197 (19)	0.2972 (2)	−0.1844 (2)	0.0148 (11)	
C9A	0.6739 (2)	0.1778 (2)	−0.0399 (3)	0.0202 (12)	
C10A	0.6657 (2)	0.1251 (2)	−0.0379 (3)	0.0286 (13)	
H10A	0.691093	0.102591	−0.059468	0.034*	
C11A	0.6204 (3)	0.1056 (2)	−0.0044 (4)	0.0401 (16)	
H11A	0.614608	0.069887	−0.003718	0.048*	
C12A	0.5843 (2)	0.1383 (2)	0.0276 (4)	0.0350 (15)	
C13A	0.5921 (2)	0.1902 (2)	0.0279 (3)	0.0271 (13)	
C14A	0.6371 (2)	0.2107 (2)	−0.0059 (3)	0.0218 (12)	
H14A	0.642506	0.246471	−0.005889	0.026*	
O2A	0.89023 (13)	0.20073 (14)	0.03517 (19)	0.0215 (8)	
C15A	0.92164 (19)	0.1904 (2)	−0.0238 (3)	0.0185 (12)	
C16A	0.96642 (19)	0.1581 (2)	−0.0071 (3)	0.0193 (12)	
H16A	0.990825	0.153465	−0.052699	0.023*	
C17A	0.9736 (2)	0.1354 (2)	0.0697 (3)	0.0226 (13)	
H17A	0.948054	0.139789	0.113545	0.027*	
C18A	1.0186 (2)	0.1035 (2)	0.0928 (3)	0.0273 (14)	
H18A	1.006874	0.069948	0.114942	0.033*	
H18B	1.039407	0.097553	0.039344	0.033*	
C19A	1.0761 (2)	0.1733 (2)	0.1246 (4)	0.0365 (15)	
H19A	1.101101	0.162373	0.080490	0.055*	
H19B	1.093584	0.191439	0.172349	0.055*	
H19C	1.051144	0.196023	0.096980	0.055*	
C20A	1.0865 (2)	0.0933 (2)	0.1990 (4)	0.0406 (15)	
H20A	1.109638	0.081026	0.152897	0.061*	
H20B	1.068379	0.064377	0.225073	0.061*	
H20C	1.106198	0.110843	0.244765	0.061*	
O1A	0.93145 (13)	0.28774 (13)	−0.2136 (2)	0.0206 (8)	
C21A	0.9410 (6)	0.3289 (5)	−0.2758 (17)	0.022 (2)	0.718 (9)
H21A	0.915222	0.328831	−0.324386	0.027*	0.718 (9)
C22A	0.9429 (4)	0.3805 (5)	−0.2302 (6)	0.028 (2)	0.718 (9)
H22A	0.913750	0.402123	−0.248009	0.034*	0.718 (9)
H22B	0.942657	0.376599	−0.165226	0.034*	0.718 (9)
C23A	0.9922 (4)	0.4027 (4)	−0.2612 (6)	0.035 (2)	0.718 (9)
H23A	0.987716	0.420478	−0.318291	0.042*	0.718 (9)
H23B	1.005922	0.427143	−0.217496	0.042*	0.718 (9)
O3A	1.0252 (2)	0.3599 (2)	−0.2706 (4)	0.0374 (16)	0.718 (9)
C24A	0.9951 (6)	0.3219 (4)	−0.3124 (14)	0.028 (2)	0.718 (9)
H24A	1.008122	0.287436	−0.298384	0.034*	0.718 (9)
H24B	0.995381	0.326476	−0.377212	0.034*	0.718 (9)
C21C	0.9418 (16)	0.3249 (13)	−0.281 (5)	0.024 (3)	0.282 (9)
H21C	0.919160	0.318416	−0.332591	0.029*	0.282 (9)
C22C	0.9382 (9)	0.3802 (12)	−0.2548 (16)	0.026 (3)	0.282 (9)
H22E	0.907338	0.395998	−0.280376	0.031*	0.282 (9)
H22F	0.936849	0.383724	−0.189820	0.031*	0.282 (9)
C23C	0.9853 (9)	0.4050 (8)	−0.2906 (15)	0.032 (3)	0.282 (9)
H23E	0.975859	0.433007	−0.331212	0.039*	0.282 (9)
H23F	1.005744	0.419462	−0.241821	0.039*	0.282 (9)
O3C	1.0136 (5)	0.3679 (5)	−0.3358 (10)	0.037 (3)	0.282 (9)
C24C	0.9974 (16)	0.3185 (9)	−0.309 (4)	0.029 (3)	0.282 (9)
H24E	1.018320	0.306284	−0.258484	0.035*	0.282 (9)
H24F	1.000348	0.293884	−0.357681	0.035*	0.282 (9)
O4A	0.99752 (15)	0.18082 (17)	0.2950 (2)	0.0274 (9)	
H4C	1.011 (2)	0.164 (2)	0.253 (3)	0.041*	
H4D	0.9765 (17)	0.2009 (18)	0.269 (3)	0.041*	
O5A	0.92301 (15)	0.23351 (18)	0.2047 (2)	0.0360 (10)	
H5C	0.912 (2)	0.226 (2)	0.154 (2)	0.054*	
H5D	0.8972 (16)	0.246 (2)	0.228 (4)	0.054*	
N6A	0.500000	0.000000	0.0706 (10)	0.100 (5)	
C25A	0.500000	0.000000	−0.0068 (10)	0.065 (5)	
C26A	0.500000	0.000000	−0.1044 (10)	0.096 (6)	
H26A	0.470993	−0.018658	−0.125464	0.144*	0.25
H26B	0.530662	−0.015792	−0.125464	0.144*	0.25
H26C	0.498346	0.034450	−0.125464	0.144*	0.25
Cl1B	0.95744 (6)	0.26892 (6)	−0.55731 (8)	0.0386 (4)	
F1B	0.96334 (12)	0.37984 (13)	−0.5399 (2)	0.0437 (9)	
N1B	0.74716 (16)	0.16513 (17)	−0.3001 (2)	0.0194 (10)	
N2B	0.80584 (16)	0.22306 (17)	−0.3705 (2)	0.0202 (10)	
N3B	0.77931 (16)	0.30174 (16)	−0.4234 (2)	0.0167 (9)	
H3B	0.753408	0.322621	−0.430670	0.020*	
C1B	0.7923 (2)	0.1797 (2)	−0.3279 (3)	0.0211 (12)	
H1B	0.819331	0.156553	−0.316512	0.025*	
C2B	0.7685 (2)	0.25621 (19)	−0.3862 (3)	0.0167 (11)	
C3B	0.71662 (19)	0.24443 (19)	−0.3633 (3)	0.0143 (11)	
C4B	0.67449 (19)	0.27542 (19)	−0.3855 (3)	0.0170 (11)	
H4B	0.679762	0.306750	−0.415510	0.020*	
N4B	0.58392 (16)	0.28961 (16)	−0.3930 (2)	0.0197 (10)	
H4B1	0.559066	0.295893	−0.355643	0.024*	
C5B	0.6261 (2)	0.2604 (2)	−0.3638 (3)	0.0175 (11)	
N5B	0.45204 (17)	0.37462 (18)	−0.6582 (2)	0.0253 (11)	
C6B	0.6177 (2)	0.2159 (2)	−0.3129 (3)	0.0223 (12)	
C7B	0.6581 (2)	0.1847 (2)	−0.2923 (3)	0.0204 (12)	
H7B	0.652526	0.154174	−0.260097	0.024*	
C8B	0.7077 (2)	0.1982 (2)	−0.3191 (3)	0.0183 (12)	
C9B	0.8275 (2)	0.3199 (2)	−0.4520 (3)	0.0176 (11)	
C10B	0.8359 (2)	0.3723 (2)	−0.4483 (3)	0.0208 (11)	
H10B	0.810418	0.394157	−0.424751	0.025*	
C11B	0.8815 (2)	0.3928 (2)	−0.4789 (3)	0.0274 (13)	
H11B	0.887333	0.428497	−0.476902	0.033*	
C12B	0.9179 (2)	0.3606 (2)	−0.5119 (3)	0.0285 (13)	
C13B	0.9105 (2)	0.3082 (2)	−0.5159 (3)	0.0243 (12)	
C14B	0.8650 (2)	0.2880 (2)	−0.4860 (3)	0.0214 (12)	
H14B	0.859325	0.252258	−0.488762	0.026*	
O2B	0.61156 (13)	0.29856 (14)	−0.53473 (19)	0.0209 (8)	
C15B	0.58026 (19)	0.3081 (2)	−0.4757 (3)	0.0170 (11)	
C16B	0.53511 (19)	0.3408 (2)	−0.4920 (3)	0.0202 (12)	
H16B	0.510440	0.344796	−0.446629	0.024*	
C17B	0.5283 (2)	0.36465 (19)	−0.5681 (3)	0.0206 (12)	
H17B	0.553618	0.360544	−0.612326	0.025*	
C18B	0.4836 (2)	0.3974 (2)	−0.5888 (3)	0.0272 (14)	
H18C	0.462781	0.402146	−0.534975	0.033*	
H18D	0.495562	0.431380	−0.608348	0.033*	
C19B	0.4253 (2)	0.3290 (2)	−0.6251 (3)	0.0325 (14)	
H19D	0.405129	0.313979	−0.672842	0.049*	
H19E	0.402741	0.338744	−0.576490	0.049*	
H19F	0.450346	0.304135	−0.604111	0.049*	
C20B	0.4154 (2)	0.4119 (2)	−0.6936 (4)	0.0407 (15)	
H20D	0.396854	0.396662	−0.743090	0.061*	
H20E	0.433746	0.442196	−0.713900	0.061*	
H20F	0.391252	0.421513	−0.647204	0.061*	
O1B	0.56935 (19)	0.2103 (2)	−0.2827 (3)	0.0193 (12)	0.787 (5)
C21B	0.5595 (2)	0.1713 (3)	−0.2175 (4)	0.0215 (14)	0.787 (5)
H21B	0.588006	0.168742	−0.173961	0.026*	0.787 (5)
C22B	0.5094 (2)	0.1847 (3)	−0.1725 (4)	0.0226 (14)	0.787 (5)
H22C	0.509418	0.173841	−0.109982	0.027*	0.787 (5)
H22D	0.502461	0.221710	−0.175706	0.027*	0.787 (5)
C23B	0.4716 (3)	0.1550 (3)	−0.2253 (5)	0.0273 (15)	0.787 (5)
H23C	0.440105	0.148926	−0.190939	0.033*	0.787 (5)
H23D	0.462678	0.173237	−0.280321	0.033*	0.787 (5)
O3B	0.49710 (19)	0.10754 (19)	−0.2447 (3)	0.0357 (13)	0.787 (5)
C24B	0.5490 (3)	0.1204 (3)	−0.2617 (5)	0.0354 (17)	0.787 (5)
H24C	0.555014	0.122991	−0.325968	0.042*	0.787 (5)
H24D	0.571903	0.093880	−0.237446	0.042*	0.787 (5)
O1D	0.5663 (7)	0.1987 (7)	−0.3159 (11)	0.025 (4)	0.213 (5)
C21D	0.5525 (7)	0.1519 (9)	−0.2718 (12)	0.026 (3)	0.213 (5)
H21E	0.570895	0.122722	−0.299762	0.032*	0.213 (5)
C22D	0.5634 (7)	0.1525 (10)	−0.1741 (12)	0.030 (3)	0.213 (5)
H22G	0.586501	0.180850	−0.158334	0.036*	0.213 (5)
H22H	0.578862	0.119913	−0.154763	0.036*	0.213 (5)
C23D	0.5114 (7)	0.1598 (9)	−0.1336 (12)	0.029 (3)	0.213 (5)
H23G	0.510087	0.144714	−0.073751	0.035*	0.213 (5)
H23H	0.502886	0.196451	−0.129409	0.035*	0.213 (5)
O3D	0.4770 (6)	0.1344 (8)	−0.1910 (11)	0.032 (3)	0.213 (5)
C24D	0.4952 (7)	0.1431 (10)	−0.2782 (12)	0.030 (3)	0.213 (5)
H24G	0.478318	0.173350	−0.304025	0.036*	0.213 (5)
H24H	0.487987	0.113276	−0.316080	0.036*	0.213 (5)
O4B	0.50270 (15)	0.32327 (17)	−0.7952 (2)	0.0268 (9)	
H4E	0.489 (2)	0.340 (2)	−0.752 (3)	0.040*	
H4F	0.5294 (15)	0.3086 (19)	−0.774 (3)	0.040*	
O5B	0.58067 (15)	0.27400 (17)	−0.7083 (2)	0.0304 (9)	
H5E	0.589 (2)	0.284 (2)	−0.657 (2)	0.046*	
H5F	0.6065 (16)	0.262 (2)	−0.736 (3)	0.046*	
N6B	1.000000	0.500000	−0.4494 (7)	0.073 (4)	
C25B	1.000000	0.500000	−0.5259 (8)	0.039 (3)	
C26B	1.000000	0.500000	−0.6212 (7)	0.046 (3)	
H26D	1.029352	0.481890	−0.642284	0.069*	0.25
H26E	1.001008	0.534475	−0.642284	0.069*	0.25
H26F	0.969640	0.483635	−0.642284	0.069*	0.25

### Atomic displacement parameters (Å^2^)

**Table:**

	*U*^11^	*U*^22^	*U*^33^	*U*^12^	*U*^13^	*U*^23^
Cl1A	0.0283 (8)	0.0446 (9)	0.0350 (6)	0.0075 (7)	0.0128 (6)	0.0002 (6)
F1A	0.040 (2)	0.046 (2)	0.076 (2)	−0.0179 (18)	0.0315 (19)	0.0005 (19)
N1A	0.016 (2)	0.020 (2)	0.0188 (17)	0.0039 (18)	0.0012 (16)	0.0001 (17)
N2A	0.019 (3)	0.022 (3)	0.0201 (18)	0.004 (2)	−0.0011 (17)	0.0057 (18)
N3A	0.015 (2)	0.020 (2)	0.0203 (17)	0.001 (2)	0.0018 (17)	0.0050 (17)
C1A	0.023 (3)	0.019 (3)	0.018 (2)	0.005 (2)	0.000 (2)	0.000 (2)
C2A	0.017 (3)	0.020 (3)	0.0126 (18)	0.002 (2)	−0.0013 (19)	−0.0014 (18)
C3A	0.017 (3)	0.019 (3)	0.015 (2)	0.002 (2)	0.0008 (19)	0.0001 (18)
C4A	0.017 (3)	0.018 (3)	0.0109 (19)	0.001 (2)	−0.0007 (18)	−0.0006 (18)
N4A	0.014 (2)	0.020 (2)	0.0154 (17)	0.0072 (19)	0.0011 (16)	0.0038 (16)
C5A	0.017 (3)	0.017 (3)	0.0150 (19)	0.007 (2)	0.0028 (18)	0.0031 (19)
N5A	0.020 (3)	0.029 (3)	0.028 (2)	0.006 (2)	−0.0035 (19)	0.009 (2)
C6A	0.015 (3)	0.017 (3)	0.018 (2)	0.004 (2)	0.0012 (19)	−0.0011 (19)
C7A	0.019 (3)	0.016 (3)	0.017 (2)	−0.002 (2)	−0.0002 (19)	0.0025 (18)
C8A	0.013 (3)	0.016 (3)	0.0152 (19)	0.003 (2)	−0.0012 (18)	−0.0030 (18)
C9A	0.019 (3)	0.026 (3)	0.0152 (19)	−0.004 (2)	0.001 (2)	0.002 (2)
C10A	0.032 (3)	0.020 (3)	0.034 (2)	0.001 (3)	0.010 (2)	−0.005 (2)
C11A	0.044 (4)	0.025 (4)	0.051 (3)	−0.012 (3)	0.019 (3)	−0.003 (3)
C12A	0.027 (3)	0.034 (4)	0.044 (3)	−0.010 (3)	0.018 (3)	0.003 (3)
C13A	0.019 (3)	0.037 (4)	0.025 (2)	0.001 (3)	0.007 (2)	0.001 (2)
C14A	0.023 (3)	0.026 (3)	0.016 (2)	−0.001 (2)	0.001 (2)	0.001 (2)
O2A	0.018 (2)	0.028 (2)	0.0180 (14)	0.0041 (17)	0.0020 (14)	0.0017 (15)
C15A	0.015 (3)	0.020 (3)	0.020 (2)	0.001 (2)	−0.001 (2)	0.001 (2)
C16A	0.020 (3)	0.023 (3)	0.0157 (19)	0.008 (2)	0.001 (2)	0.001 (2)
C17A	0.017 (3)	0.030 (3)	0.021 (2)	0.004 (3)	0.002 (2)	0.004 (2)
C18A	0.027 (3)	0.031 (4)	0.024 (2)	0.011 (3)	−0.003 (2)	0.005 (2)
C19A	0.021 (3)	0.047 (4)	0.041 (3)	0.000 (3)	0.002 (3)	0.019 (3)
C20A	0.033 (4)	0.044 (4)	0.045 (3)	0.009 (3)	−0.010 (3)	0.017 (3)
O1A	0.0143 (19)	0.022 (2)	0.0254 (16)	0.0020 (16)	0.0037 (14)	0.0069 (15)
C21A	0.017 (4)	0.023 (4)	0.027 (4)	−0.002 (3)	0.006 (3)	0.009 (4)
C22A	0.027 (4)	0.022 (4)	0.035 (4)	0.000 (3)	0.000 (3)	0.003 (4)
C23A	0.023 (4)	0.032 (4)	0.050 (5)	−0.003 (3)	−0.002 (4)	−0.002 (4)
O3A	0.023 (3)	0.031 (3)	0.058 (3)	−0.001 (2)	−0.001 (2)	−0.006 (3)
C24A	0.023 (4)	0.030 (4)	0.031 (4)	0.003 (3)	0.007 (3)	0.006 (3)
C21C	0.022 (6)	0.021 (6)	0.028 (6)	0.002 (6)	0.005 (6)	0.005 (6)
C22C	0.021 (6)	0.022 (6)	0.035 (7)	0.005 (5)	0.000 (6)	0.009 (6)
C23C	0.024 (6)	0.030 (6)	0.043 (7)	0.002 (5)	−0.001 (6)	0.006 (6)
O3C	0.025 (5)	0.035 (5)	0.050 (5)	−0.003 (5)	0.012 (5)	0.004 (5)
C24C	0.022 (6)	0.030 (6)	0.035 (6)	0.000 (6)	0.008 (6)	0.004 (6)
O4A	0.024 (2)	0.036 (3)	0.0221 (16)	−0.0015 (18)	−0.0012 (17)	0.0049 (16)
O5A	0.025 (2)	0.061 (3)	0.0220 (17)	0.013 (2)	0.0019 (16)	−0.0072 (19)
N6A	0.108 (9)	0.108 (9)	0.084 (10)	0.000	0.000	0.000
C25A	0.068 (8)	0.068 (8)	0.060 (9)	0.000	0.000	0.000
C26A	0.110 (10)	0.110 (10)	0.067 (10)	0.000	0.000	0.000
Cl1B	0.0316 (8)	0.0372 (9)	0.0470 (7)	0.0096 (7)	0.0191 (6)	0.0103 (6)
F1B	0.026 (2)	0.044 (2)	0.0611 (19)	−0.0100 (16)	0.0132 (16)	0.0082 (17)
N1B	0.018 (3)	0.022 (2)	0.0182 (17)	0.0003 (19)	−0.0031 (16)	0.0021 (17)
N2B	0.016 (2)	0.021 (3)	0.0237 (19)	0.0019 (19)	0.0009 (17)	0.0036 (18)
N3B	0.014 (2)	0.015 (2)	0.0209 (17)	−0.0010 (19)	0.0018 (16)	0.0016 (17)
C1B	0.016 (3)	0.022 (3)	0.025 (2)	0.003 (2)	−0.002 (2)	0.007 (2)
C2B	0.018 (3)	0.019 (3)	0.0125 (18)	0.001 (2)	−0.0006 (19)	−0.0004 (18)
C3B	0.016 (3)	0.014 (3)	0.013 (2)	0.000 (2)	−0.0005 (18)	−0.0001 (17)
C4B	0.018 (3)	0.016 (3)	0.017 (2)	−0.001 (2)	−0.0005 (19)	0.0025 (19)
N4B	0.015 (2)	0.028 (3)	0.0164 (17)	0.005 (2)	0.0022 (17)	0.0016 (17)
C5B	0.019 (3)	0.018 (3)	0.015 (2)	0.004 (2)	−0.0008 (19)	−0.0008 (19)
N5B	0.022 (3)	0.031 (3)	0.0231 (19)	0.007 (2)	−0.0067 (19)	0.0055 (19)
C6B	0.018 (3)	0.026 (3)	0.023 (2)	−0.005 (2)	0.003 (2)	0.005 (2)
C7B	0.019 (3)	0.022 (3)	0.020 (2)	0.001 (2)	0.002 (2)	0.005 (2)
C8B	0.021 (3)	0.019 (3)	0.0145 (19)	0.004 (2)	0.0005 (19)	0.0056 (19)
C9B	0.018 (3)	0.018 (3)	0.017 (2)	−0.002 (2)	0.0013 (19)	0.0031 (19)
C10B	0.019 (3)	0.020 (3)	0.024 (2)	−0.003 (2)	−0.0003 (19)	0.0019 (19)
C11B	0.029 (3)	0.020 (3)	0.033 (2)	−0.007 (2)	0.003 (2)	0.003 (2)
C12B	0.023 (3)	0.033 (4)	0.030 (2)	−0.009 (3)	0.004 (2)	0.004 (2)
C13B	0.026 (3)	0.023 (3)	0.024 (2)	0.005 (2)	0.003 (2)	0.005 (2)
C14B	0.024 (3)	0.020 (3)	0.020 (2)	−0.001 (2)	0.001 (2)	0.002 (2)
O2B	0.015 (2)	0.030 (2)	0.0173 (14)	0.0087 (16)	0.0007 (14)	−0.0002 (15)
C15B	0.012 (3)	0.020 (3)	0.019 (2)	−0.001 (2)	−0.0009 (19)	0.000 (2)
C16B	0.016 (3)	0.024 (3)	0.020 (2)	0.004 (2)	0.001 (2)	0.000 (2)
C17B	0.020 (3)	0.019 (3)	0.023 (2)	0.004 (2)	−0.005 (2)	−0.003 (2)
C18B	0.027 (3)	0.030 (4)	0.024 (2)	0.011 (3)	−0.003 (2)	−0.001 (2)
C19B	0.024 (3)	0.036 (4)	0.038 (3)	0.002 (3)	−0.007 (3)	0.009 (3)
C20B	0.037 (4)	0.043 (4)	0.042 (3)	0.016 (3)	−0.014 (3)	0.009 (3)
O1B	0.013 (2)	0.023 (3)	0.022 (3)	0.001 (2)	0.006 (2)	0.008 (2)
C21B	0.019 (3)	0.022 (3)	0.024 (3)	0.002 (3)	−0.002 (2)	0.010 (3)
C22B	0.020 (3)	0.026 (3)	0.022 (3)	−0.002 (3)	0.000 (2)	0.005 (2)
C23B	0.023 (3)	0.028 (4)	0.032 (3)	−0.004 (3)	0.003 (3)	−0.003 (3)
O3B	0.029 (3)	0.025 (3)	0.053 (3)	−0.006 (2)	0.008 (2)	−0.004 (2)
C24B	0.026 (4)	0.033 (4)	0.047 (3)	0.002 (3)	0.014 (3)	0.000 (3)
O1D	0.025 (6)	0.023 (7)	0.027 (7)	0.001 (6)	0.007 (6)	0.006 (6)
C21D	0.019 (5)	0.027 (5)	0.033 (5)	−0.003 (5)	0.005 (5)	0.005 (5)
C22D	0.023 (6)	0.033 (6)	0.035 (6)	−0.005 (6)	0.000 (6)	0.006 (6)
C23D	0.024 (6)	0.032 (6)	0.031 (6)	−0.003 (6)	−0.004 (6)	0.001 (6)
O3D	0.024 (6)	0.036 (6)	0.036 (6)	−0.005 (5)	0.001 (5)	0.003 (5)
C24D	0.022 (6)	0.031 (6)	0.037 (6)	−0.001 (5)	−0.002 (5)	0.005 (5)
O4B	0.019 (2)	0.039 (3)	0.0230 (16)	−0.0001 (18)	−0.0041 (17)	−0.0031 (16)
O5B	0.021 (2)	0.047 (3)	0.0224 (17)	0.0058 (19)	−0.0002 (15)	−0.0103 (17)
N6B	0.086 (7)	0.086 (7)	0.048 (7)	0.000	0.000	0.000
C25B	0.037 (5)	0.037 (5)	0.043 (6)	0.000	0.000	0.000
C26B	0.050 (5)	0.050 (5)	0.039 (6)	0.000	0.000	0.000

### Geometric parameters (Å, º)

**Table:**

Cl1A—C13A	1.729 (6)	F1B—C12B	1.362 (6)
F1A—C12A	1.349 (6)	N1B—C1B	1.315 (6)
N1A—C1A	1.315 (6)	N1B—C8B	1.380 (6)
N1A—C8A	1.377 (6)	N2B—C2B	1.332 (6)
N2A—C2A	1.329 (6)	N2B—C1B	1.355 (6)
N2A—C1A	1.340 (6)	N3B—C2B	1.351 (6)
N3A—C2A	1.368 (6)	N3B—C9B	1.420 (6)
N3A—C9A	1.407 (6)	N3B—H3B	0.8800
N3A—H3A	0.8800	C1B—H1B	0.9500
C1A—H1A	0.9500	C2B—C3B	1.439 (7)
C2A—C3A	1.437 (7)	C3B—C8B	1.407 (6)
C3A—C4A	1.394 (6)	C3B—C4B	1.413 (7)
C3A—C8A	1.415 (6)	C4B—C5B	1.369 (7)
C4A—C5A	1.369 (6)	C4B—H4B	0.9500
C4A—H4A	0.9500	N4B—C15B	1.348 (5)
N4A—C15A	1.356 (5)	N4B—C5B	1.417 (6)
N4A—C5A	1.411 (6)	N4B—H4B1	0.8800
N4A—H4A1	0.8800	C5B—C6B	1.418 (7)
C5A—C6A	1.424 (6)	N5B—C18B	1.466 (6)
N5A—C20A	1.454 (7)	N5B—C20B	1.472 (7)
N5A—C19A	1.467 (6)	N5B—C19B	1.474 (6)
N5A—C18A	1.472 (6)	C6B—O1B	1.358 (7)
C6A—O1A	1.363 (5)	C6B—C7B	1.375 (7)
C6A—C7A	1.373 (6)	C6B—O1D	1.424 (17)
C7A—C8A	1.411 (7)	C7B—C8B	1.410 (7)
C7A—H7A	0.9500	C7B—H7B	0.9500
C9A—C14A	1.396 (7)	C9B—C14B	1.390 (7)
C9A—C10A	1.399 (7)	C9B—C10B	1.396 (7)
C10A—C11A	1.389 (8)	C10B—C11B	1.391 (7)
C10A—H10A	0.9500	C10B—H10B	0.9500
C11A—C12A	1.367 (8)	C11B—C12B	1.370 (7)
C11A—H11A	0.9500	C11B—H11B	0.9500
C12A—C13A	1.378 (8)	C12B—C13B	1.391 (8)
C13A—C14A	1.393 (7)	C13B—C14B	1.383 (7)
C14A—H14A	0.9500	C14B—H14B	0.9500
O2A—C15A	1.246 (6)	O2B—C15B	1.241 (5)
C15A—C16A	1.471 (7)	C15B—C16B	1.483 (7)
C16A—C17A	1.322 (6)	C16B—C17B	1.325 (6)
C16A—H16A	0.9500	C16B—H16B	0.9500
C17A—C18A	1.491 (7)	C17B—C18B	1.489 (7)
C17A—H17A	0.9500	C17B—H17B	0.9500
C18A—H18A	0.9900	C18B—H18C	0.9900
C18A—H18B	0.9900	C18B—H18D	0.9900
C19A—H19A	0.9800	C19B—H19D	0.9800
C19A—H19B	0.9800	C19B—H19E	0.9800
C19A—H19C	0.9800	C19B—H19F	0.9800
C20A—H20A	0.9800	C20B—H20D	0.9800
C20A—H20B	0.9800	C20B—H20E	0.9800
C20A—H20C	0.9800	C20B—H20F	0.9800
O1A—C21C	1.43 (6)	O1B—C21B	1.445 (7)
O1A—C21A	1.456 (19)	C21B—C24B	1.520 (9)
C21A—C22A	1.522 (12)	C21B—C22B	1.521 (8)
C21A—C24A	1.534 (10)	C21B—H21B	1.0000
C21A—H21A	1.0000	C22B—C23B	1.495 (8)
C22A—C23A	1.495 (10)	C22B—H22C	0.9900
C22A—H22A	0.9900	C22B—H22D	0.9900
C22A—H22B	0.9900	C23B—O3B	1.444 (8)
C23A—O3A	1.426 (9)	C23B—H23C	0.9900
C23A—H23A	0.9900	C23B—H23D	0.9900
C23A—H23B	0.9900	O3B—C24B	1.427 (8)
O3A—C24A	1.423 (15)	C24B—H24C	0.9900
C24A—H24A	0.9900	C24B—H24D	0.9900
C24A—H24B	0.9900	O1D—C21D	1.445 (19)
C21C—C22C	1.51 (2)	C21D—C22D	1.508 (19)
C21C—C24C	1.53 (2)	C21D—C24D	1.524 (19)
C21C—H21C	1.0000	C21D—H21E	1.0000
C22C—C23C	1.497 (19)	C22D—C23D	1.509 (19)
C22C—H22E	0.9900	C22D—H22G	0.9900
C22C—H22F	0.9900	C22D—H22H	0.9900
C23C—O3C	1.403 (18)	C23D—O3D	1.420 (18)
C23C—H23E	0.9900	C23D—H23G	0.9900
C23C—H23F	0.9900	C23D—H23H	0.9900
O3C—C24C	1.43 (2)	O3D—C24D	1.424 (18)
C24C—H24E	0.9900	C24D—H24G	0.9900
C24C—H24F	0.9900	C24D—H24H	0.9900
O4A—H4C	0.87 (3)	O4B—H4E	0.87 (3)
O4A—H4D	0.86 (3)	O4B—H4F	0.86 (3)
O5A—H5C	0.84 (3)	O5B—H5E	0.85 (3)
O5A—H5D	0.84 (3)	O5B—H5F	0.85 (3)
N6A—C25A	1.174 (17)	N6B—C25B	1.161 (14)
C25A—C26A	1.48 (2)	C25B—C26B	1.444 (14)
C26A—H26A	0.9600	C26B—H26D	0.9600
C26A—H26B	0.9600	C26B—H26E	0.9600
C26A—H26C	0.9600	C26B—H26F	0.9600
C26A—H26A^i^	0.9600	C26B—H26D^iv^	0.9600
C26A—H26A^ii^	0.9600	C26B—H26D^v^	0.9600
C26A—H26A^iii^	0.9600	C26B—H26D^vi^	0.9600
C26A—H26B^i^	0.9600	C26B—H26E^iv^	0.9600
C26A—H26B^ii^	0.9600	C26B—H26E^v^	0.9600
C26A—H26B^iii^	0.9600	C26B—H26E^vi^	0.9600
C26A—H26C^i^	0.9599	C26B—H26F^iv^	0.9599
C26A—H26C^ii^	0.9599	C26B—H26F^v^	0.9599
C26A—H26C^iii^	0.9599	C26B—H26F^vi^	0.9599
Cl1B—C13B	1.725 (5)		
			
C1A—N1A—C8A	115.3 (4)	C2B—N2B—C1B	116.1 (4)
C2A—N2A—C1A	116.2 (4)	C2B—N3B—C9B	127.7 (5)
C2A—N3A—C9A	128.6 (5)	C2B—N3B—H3B	116.2
C2A—N3A—H3A	115.7	C9B—N3B—H3B	116.2
C9A—N3A—H3A	115.7	N1B—C1B—N2B	129.3 (5)
N1A—C1A—N2A	129.3 (5)	N1B—C1B—H1B	115.3
N1A—C1A—H1A	115.3	N2B—C1B—H1B	115.3
N2A—C1A—H1A	115.3	N2B—C2B—N3B	119.8 (5)
N2A—C2A—N3A	119.1 (5)	N2B—C2B—C3B	120.9 (5)
N2A—C2A—C3A	121.5 (4)	N3B—C2B—C3B	119.3 (5)
N3A—C2A—C3A	119.4 (5)	C8B—C3B—C4B	118.7 (5)
C4A—C3A—C8A	118.9 (5)	C8B—C3B—C2B	117.2 (5)
C4A—C3A—C2A	124.8 (5)	C4B—C3B—C2B	124.0 (4)
C8A—C3A—C2A	116.3 (4)	C5B—C4B—C3B	120.2 (5)
C5A—C4A—C3A	121.6 (5)	C5B—C4B—H4B	119.9
C5A—C4A—H4A	119.2	C3B—C4B—H4B	119.9
C3A—C4A—H4A	119.2	C15B—N4B—C5B	122.8 (4)
C15A—N4A—C5A	122.5 (4)	C15B—N4B—H4B1	118.6
C15A—N4A—H4A1	118.7	C5B—N4B—H4B1	118.6
C5A—N4A—H4A1	118.7	C4B—C5B—N4B	119.6 (5)
C4A—C5A—N4A	121.2 (4)	C4B—C5B—C6B	120.7 (5)
C4A—C5A—C6A	119.2 (5)	N4B—C5B—C6B	119.6 (5)
N4A—C5A—C6A	119.6 (4)	C18B—N5B—C20B	111.0 (4)
C20A—N5A—C19A	110.4 (4)	C18B—N5B—C19B	110.8 (4)
C20A—N5A—C18A	111.4 (4)	C20B—N5B—C19B	110.6 (4)
C19A—N5A—C18A	111.1 (4)	O1B—C6B—C7B	125.4 (5)
O1A—C6A—C7A	125.5 (5)	O1B—C6B—C5B	114.8 (5)
O1A—C6A—C5A	114.1 (4)	C7B—C6B—C5B	119.7 (5)
C7A—C6A—C5A	120.4 (5)	C7B—C6B—O1D	123.2 (9)
C6A—C7A—C8A	119.9 (5)	C5B—C6B—O1D	113.0 (9)
C6A—C7A—H7A	120.1	C6B—C7B—C8B	119.8 (5)
C8A—C7A—H7A	120.1	C6B—C7B—H7B	120.1
N1A—C8A—C7A	119.1 (4)	C8B—C7B—H7B	120.1
N1A—C8A—C3A	121.2 (5)	N1B—C8B—C3B	121.2 (5)
C7A—C8A—C3A	119.7 (5)	N1B—C8B—C7B	118.4 (4)
C14A—C9A—C10A	119.7 (5)	C3B—C8B—C7B	120.4 (5)
C14A—C9A—N3A	122.8 (5)	C14B—C9B—C10B	119.8 (5)
C10A—C9A—N3A	117.4 (5)	C14B—C9B—N3B	122.8 (5)
C11A—C10A—C9A	120.3 (5)	C10B—C9B—N3B	117.3 (5)
C11A—C10A—H10A	119.8	C11B—C10B—C9B	120.2 (5)
C9A—C10A—H10A	119.8	C11B—C10B—H10B	119.9
C12A—C11A—C10A	119.4 (6)	C9B—C10B—H10B	119.9
C12A—C11A—H11A	120.3	C12B—C11B—C10B	119.0 (5)
C10A—C11A—H11A	120.3	C12B—C11B—H11B	120.5
F1A—C12A—C11A	119.2 (6)	C10B—C11B—H11B	120.5
F1A—C12A—C13A	119.6 (5)	F1B—C12B—C11B	119.7 (5)
C11A—C12A—C13A	121.3 (5)	F1B—C12B—C13B	118.4 (5)
C12A—C13A—C14A	120.4 (5)	C11B—C12B—C13B	121.8 (5)
C12A—C13A—Cl1A	119.9 (4)	C14B—C13B—C12B	119.1 (5)
C14A—C13A—Cl1A	119.7 (4)	C14B—C13B—Cl1B	120.5 (4)
C13A—C14A—C9A	118.9 (5)	C12B—C13B—Cl1B	120.4 (4)
C13A—C14A—H14A	120.5	C13B—C14B—C9B	120.1 (5)
C9A—C14A—H14A	120.5	C13B—C14B—H14B	120.0
O2A—C15A—N4A	123.0 (5)	C9B—C14B—H14B	120.0
O2A—C15A—C16A	122.0 (4)	O2B—C15B—N4B	123.4 (5)
N4A—C15A—C16A	114.9 (4)	O2B—C15B—C16B	121.7 (4)
C17A—C16A—C15A	121.6 (5)	N4B—C15B—C16B	114.9 (4)
C17A—C16A—H16A	119.2	C17B—C16B—C15B	121.7 (4)
C15A—C16A—H16A	119.2	C17B—C16B—H16B	119.2
C16A—C17A—C18A	124.9 (5)	C15B—C16B—H16B	119.2
C16A—C17A—H17A	117.5	C16B—C17B—C18B	124.2 (5)
C18A—C17A—H17A	117.5	C16B—C17B—H17B	117.9
N5A—C18A—C17A	110.8 (5)	C18B—C17B—H17B	117.9
N5A—C18A—H18A	109.5	N5B—C18B—C17B	111.1 (4)
C17A—C18A—H18A	109.5	N5B—C18B—H18C	109.4
N5A—C18A—H18B	109.5	C17B—C18B—H18C	109.4
C17A—C18A—H18B	109.5	N5B—C18B—H18D	109.4
H18A—C18A—H18B	108.1	C17B—C18B—H18D	109.4
N5A—C19A—H19A	109.5	H18C—C18B—H18D	108.0
N5A—C19A—H19B	109.5	N5B—C19B—H19D	109.5
H19A—C19A—H19B	109.5	N5B—C19B—H19E	109.5
N5A—C19A—H19C	109.5	H19D—C19B—H19E	109.5
H19A—C19A—H19C	109.5	N5B—C19B—H19F	109.5
H19B—C19A—H19C	109.5	H19D—C19B—H19F	109.5
N5A—C20A—H20A	109.5	H19E—C19B—H19F	109.5
N5A—C20A—H20B	109.5	N5B—C20B—H20D	109.5
H20A—C20A—H20B	109.5	N5B—C20B—H20E	109.5
N5A—C20A—H20C	109.5	H20D—C20B—H20E	109.5
H20A—C20A—H20C	109.5	N5B—C20B—H20F	109.5
H20B—C20A—H20C	109.5	H20D—C20B—H20F	109.5
C6A—O1A—C21C	118.2 (19)	H20E—C20B—H20F	109.5
C6A—O1A—C21A	116.7 (8)	C6B—O1B—C21B	118.4 (5)
O1A—C21A—C22A	111.8 (16)	O1B—C21B—C24B	110.6 (6)
O1A—C21A—C24A	107.7 (13)	O1B—C21B—C22B	107.4 (5)
C22A—C21A—C24A	103.9 (9)	C24B—C21B—C22B	104.1 (5)
O1A—C21A—H21A	111.0	O1B—C21B—H21B	111.5
C22A—C21A—H21A	111.0	C24B—C21B—H21B	111.5
C24A—C21A—H21A	111.0	C22B—C21B—H21B	111.5
C23A—C22A—C21A	103.4 (7)	C23B—C22B—C21B	102.3 (5)
C23A—C22A—H22A	111.1	C23B—C22B—H22C	111.3
C21A—C22A—H22A	111.1	C21B—C22B—H22C	111.3
C23A—C22A—H22B	111.1	C23B—C22B—H22D	111.3
C21A—C22A—H22B	111.1	C21B—C22B—H22D	111.3
H22A—C22A—H22B	109.0	H22C—C22B—H22D	109.2
O3A—C23A—C22A	104.5 (7)	O3B—C23B—C22B	104.5 (5)
O3A—C23A—H23A	110.8	O3B—C23B—H23C	110.8
C22A—C23A—H23A	110.8	C22B—C23B—H23C	110.8
O3A—C23A—H23B	110.8	O3B—C23B—H23D	110.8
C22A—C23A—H23B	110.8	C22B—C23B—H23D	110.8
H23A—C23A—H23B	108.9	H23C—C23B—H23D	108.9
C24A—O3A—C23A	105.0 (8)	C24B—O3B—C23B	106.0 (5)
O3A—C24A—C21A	105.6 (11)	O3B—C24B—C21B	107.5 (5)
O3A—C24A—H24A	110.6	O3B—C24B—H24C	110.2
C21A—C24A—H24A	110.6	C21B—C24B—H24C	110.2
O3A—C24A—H24B	110.6	O3B—C24B—H24D	110.2
C21A—C24A—H24B	110.6	C21B—C24B—H24D	110.2
H24A—C24A—H24B	108.7	H24C—C24B—H24D	108.5
O1A—C21C—C22C	117 (4)	C6B—O1D—C21D	119.5 (15)
O1A—C21C—C24C	108 (4)	O1D—C21D—C22D	113.5 (19)
C22C—C21C—C24C	103.6 (18)	O1D—C21D—C24D	110.3 (17)
O1A—C21C—H21C	109.4	C22D—C21D—C24D	104.5 (13)
C22C—C21C—H21C	109.4	O1D—C21D—H21E	109.4
C24C—C21C—H21C	109.4	C22D—C21D—H21E	109.4
C23C—C22C—C21C	105.9 (16)	C24D—C21D—H21E	109.4
C23C—C22C—H22E	110.6	C21D—C22D—C23D	103.3 (14)
C21C—C22C—H22E	110.6	C21D—C22D—H22G	111.1
C23C—C22C—H22F	110.6	C23D—C22D—H22G	111.1
C21C—C22C—H22F	110.6	C21D—C22D—H22H	111.1
H22E—C22C—H22F	108.7	C23D—C22D—H22H	111.1
O3C—C23C—C22C	108.2 (15)	H22G—C22D—H22H	109.1
O3C—C23C—H23E	110.0	O3D—C23D—C22D	105.4 (14)
C22C—C23C—H23E	110.0	O3D—C23D—H23G	110.7
O3C—C23C—H23F	110.0	C22D—C23D—H23G	110.7
C22C—C23C—H23F	110.0	O3D—C23D—H23H	110.7
H23E—C23C—H23F	108.4	C22D—C23D—H23H	110.7
C23C—O3C—C24C	109.3 (17)	H23G—C23D—H23H	108.8
O3C—C24C—C21C	105 (2)	C23D—O3D—C24D	106.3 (14)
O3C—C24C—H24E	110.7	O3D—C24D—C21D	107.2 (14)
C21C—C24C—H24E	110.7	O3D—C24D—H24G	110.3
O3C—C24C—H24F	110.7	C21D—C24D—H24G	110.3
C21C—C24C—H24F	110.7	O3D—C24D—H24H	110.3
H24E—C24C—H24F	108.8	C21D—C24D—H24H	110.3
H4C—O4A—H4D	104 (5)	H24G—C24D—H24H	108.5
H5C—O5A—H5D	101 (6)	H4E—O4B—H4F	107 (5)
N6A—C25A—C26A	180.0	H5E—O5B—H5F	110 (5)
C25A—C26A—H26A	109.5	N6B—C25B—C26B	180.0
C25A—C26A—H26B	109.5	C25B—C26B—H26D	109.5
H26A—C26A—H26B	109.5	C25B—C26B—H26E	109.5
C25A—C26A—H26C	109.5	H26D—C26B—H26E	109.5
H26A—C26A—H26C	109.5	C25B—C26B—H26F	109.5
H26B—C26A—H26C	109.5	H26D—C26B—H26F	109.5
C25A—C26A—H26A^i^	109.471 (1)	H26E—C26B—H26F	109.5
C25A—C26A—H26A^ii^	109.5	C25B—C26B—H26D^iv^	109.469 (1)
C25A—C26A—H26A^iii^	109.471 (1)	C25B—C26B—H26D^v^	109.469 (1)
C25A—C26A—H26B^i^	109.5	C25B—C26B—H26D^vi^	109.469 (2)
H26A^i^—C26A—H26B^i^	109.5	C25B—C26B—H26E^iv^	109.467 (2)
C25A—C26A—H26B^ii^	109.471 (1)	H26D^iv^—C26B—H26E^iv^	109.5
H26A^ii^—C26A—H26B^ii^	109.5	C25B—C26B—H26E^v^	109.467 (1)
C25A—C26A—H26B^iii^	109.471 (1)	H26D^v^—C26B—H26E^v^	109.5
H26A^iii^—C26A—H26B^iii^	109.5	C25B—C26B—H26E^vi^	109.467 (1)
C25A—C26A—H26C^i^	109.473 (1)	H26D^vi^—C26B—H26E^vi^	109.5
H26A^i^—C26A—H26C^i^	109.5	C25B—C26B—H26F^iv^	109.470 (1)
H26B^i^—C26A—H26C^i^	109.5	H26D^iv^—C26B—H26F^iv^	109.5
C25A—C26A—H26C^ii^	109.5	H26E^iv^—C26B—H26F^iv^	109.5
H26A^ii^—C26A—H26C^ii^	109.5	C25B—C26B—H26F^v^	109.470 (2)
H26B^ii^—C26A—H26C^ii^	109.5	H26D^v^—C26B—H26F^v^	109.5
C25A—C26A—H26C^iii^	109.5	H26E^v^—C26B—H26F^v^	109.5
H26A^iii^—C26A—H26C^iii^	109.5	C25B—C26B—H26F^vi^	109.470 (1)
H26B^iii^—C26A—H26C^iii^	109.5	H26D^vi^—C26B—H26F^vi^	109.5
C1B—N1B—C8B	115.2 (4)	H26E^vi^—C26B—H26F^vi^	109.5
			
C8A—N1A—C1A—N2A	−3.0 (7)	C1B—N2B—C2B—N3B	175.9 (4)
C2A—N2A—C1A—N1A	0.3 (7)	C1B—N2B—C2B—C3B	−3.6 (6)
C1A—N2A—C2A—N3A	−176.9 (4)	C9B—N3B—C2B—N2B	2.0 (7)
C1A—N2A—C2A—C3A	3.0 (6)	C9B—N3B—C2B—C3B	−178.6 (4)
C9A—N3A—C2A—N2A	−3.6 (7)	N2B—C2B—C3B—C8B	3.9 (6)
C9A—N3A—C2A—C3A	176.5 (4)	N3B—C2B—C3B—C8B	−175.6 (4)
N2A—C2A—C3A—C4A	173.4 (4)	N2B—C2B—C3B—C4B	−173.7 (4)
N3A—C2A—C3A—C4A	−6.6 (7)	N3B—C2B—C3B—C4B	6.9 (6)
N2A—C2A—C3A—C8A	−3.4 (6)	C8B—C3B—C4B—C5B	0.2 (6)
N3A—C2A—C3A—C8A	176.5 (4)	C2B—C3B—C4B—C5B	177.7 (4)
C8A—C3A—C4A—C5A	−0.2 (7)	C3B—C4B—C5B—N4B	−174.6 (4)
C2A—C3A—C4A—C5A	−177.0 (4)	C3B—C4B—C5B—C6B	5.2 (7)
C3A—C4A—C5A—N4A	175.0 (4)	C15B—N4B—C5B—C4B	43.7 (7)
C3A—C4A—C5A—C6A	−4.5 (7)	C15B—N4B—C5B—C6B	−136.0 (5)
C15A—N4A—C5A—C4A	−45.2 (7)	C4B—C5B—C6B—O1B	168.8 (5)
C15A—N4A—C5A—C6A	134.4 (5)	N4B—C5B—C6B—O1B	−11.5 (7)
C4A—C5A—C6A—O1A	−174.7 (4)	C4B—C5B—C6B—C7B	−6.5 (7)
N4A—C5A—C6A—O1A	5.8 (6)	N4B—C5B—C6B—C7B	173.2 (4)
C4A—C5A—C6A—C7A	5.1 (7)	C4B—C5B—C6B—O1D	−164.5 (9)
N4A—C5A—C6A—C7A	−174.4 (4)	N4B—C5B—C6B—O1D	15.2 (10)
O1A—C6A—C7A—C8A	178.8 (4)	O1B—C6B—C7B—C8B	−172.3 (5)
C5A—C6A—C7A—C8A	−1.0 (7)	C5B—C6B—C7B—C8B	2.4 (7)
C1A—N1A—C8A—C7A	−178.5 (4)	O1D—C6B—C7B—C8B	158.1 (9)
C1A—N1A—C8A—C3A	2.3 (6)	C1B—N1B—C8B—C3B	−1.4 (6)
C6A—C7A—C8A—N1A	177.1 (4)	C1B—N1B—C8B—C7B	179.2 (4)
C6A—C7A—C8A—C3A	−3.8 (6)	C4B—C3B—C8B—N1B	176.5 (4)
C4A—C3A—C8A—N1A	−176.4 (4)	C2B—C3B—C8B—N1B	−1.2 (6)
C2A—C3A—C8A—N1A	0.6 (6)	C4B—C3B—C8B—C7B	−4.2 (6)
C4A—C3A—C8A—C7A	4.4 (6)	C2B—C3B—C8B—C7B	178.1 (4)
C2A—C3A—C8A—C7A	−178.6 (4)	C6B—C7B—C8B—N1B	−177.8 (4)
C2A—N3A—C9A—C14A	−31.5 (7)	C6B—C7B—C8B—C3B	2.9 (7)
C2A—N3A—C9A—C10A	151.7 (5)	C2B—N3B—C9B—C14B	32.6 (7)
C14A—C9A—C10A—C11A	1.7 (8)	C2B—N3B—C9B—C10B	−149.8 (4)
N3A—C9A—C10A—C11A	178.6 (5)	C14B—C9B—C10B—C11B	0.4 (7)
C9A—C10A—C11A—C12A	−0.8 (9)	N3B—C9B—C10B—C11B	−177.3 (4)
C10A—C11A—C12A—F1A	178.5 (5)	C9B—C10B—C11B—C12B	−0.5 (7)
C10A—C11A—C12A—C13A	−0.5 (9)	C10B—C11B—C12B—F1B	−178.2 (4)
F1A—C12A—C13A—C14A	−178.0 (5)	C10B—C11B—C12B—C13B	0.2 (8)
C11A—C12A—C13A—C14A	1.0 (9)	F1B—C12B—C13B—C14B	178.7 (4)
F1A—C12A—C13A—Cl1A	2.0 (8)	C11B—C12B—C13B—C14B	0.3 (8)
C11A—C12A—C13A—Cl1A	−179.0 (5)	F1B—C12B—C13B—Cl1B	−1.2 (7)
C12A—C13A—C14A—C9A	−0.1 (7)	C11B—C12B—C13B—Cl1B	−179.6 (4)
Cl1A—C13A—C14A—C9A	179.9 (4)	C12B—C13B—C14B—C9B	−0.4 (7)
C10A—C9A—C14A—C13A	−1.2 (7)	Cl1B—C13B—C14B—C9B	179.5 (4)
N3A—C9A—C14A—C13A	−177.9 (4)	C10B—C9B—C14B—C13B	0.1 (7)
C5A—N4A—C15A—O2A	−3.5 (8)	N3B—C9B—C14B—C13B	177.6 (4)
C5A—N4A—C15A—C16A	175.4 (4)	C5B—N4B—C15B—O2B	4.9 (8)
O2A—C15A—C16A—C17A	5.0 (8)	C5B—N4B—C15B—C16B	−175.8 (4)
N4A—C15A—C16A—C17A	−173.9 (5)	O2B—C15B—C16B—C17B	−5.6 (8)
C15A—C16A—C17A—C18A	−178.0 (5)	N4B—C15B—C16B—C17B	175.2 (5)
C20A—N5A—C18A—C17A	166.2 (4)	C15B—C16B—C17B—C18B	179.2 (5)
C19A—N5A—C18A—C17A	−70.3 (5)	C20B—N5B—C18B—C17B	−166.4 (5)
C16A—C17A—C18A—N5A	112.2 (6)	C19B—N5B—C18B—C17B	70.3 (6)
C7A—C6A—O1A—C21C	−12 (2)	C16B—C17B—C18B—N5B	−113.4 (6)
C5A—C6A—O1A—C21C	167 (2)	C7B—C6B—O1B—C21B	6.0 (8)
C7A—C6A—O1A—C21A	−6.8 (11)	C5B—C6B—O1B—C21B	−168.9 (5)
C5A—C6A—O1A—C21A	173.0 (10)	C6B—O1B—C21B—C24B	−86.5 (7)
C6A—O1A—C21A—C22A	81.6 (14)	C6B—O1B—C21B—C22B	160.5 (5)
C6A—O1A—C21A—C24A	−164.8 (10)	O1B—C21B—C22B—C23B	93.5 (6)
O1A—C21A—C22A—C23A	128.0 (11)	C24B—C21B—C22B—C23B	−23.8 (7)
C24A—C21A—C22A—C23A	12.1 (18)	C21B—C22B—C23B—O3B	38.2 (6)
C21A—C22A—C23A—O3A	−33.6 (13)	C22B—C23B—O3B—C24B	−38.4 (7)
C22A—C23A—O3A—C24A	43.4 (10)	C23B—O3B—C24B—C21B	22.5 (7)
C23A—O3A—C24A—C21A	−35.0 (15)	O1B—C21B—C24B—O3B	−113.5 (6)
O1A—C21A—C24A—O3A	−105.5 (13)	C22B—C21B—C24B—O3B	1.6 (7)
C22A—C21A—C24A—O3A	13.2 (19)	C7B—C6B—O1D—C21D	19 (2)
C6A—O1A—C21C—C22C	75 (4)	C5B—C6B—O1D—C21D	176.1 (14)
C6A—O1A—C21C—C24C	−168 (2)	C6B—O1D—C21D—C22D	59 (2)
O1A—C21C—C22C—C23C	133 (3)	C6B—O1D—C21D—C24D	175.9 (15)
C24C—C21C—C22C—C23C	15 (5)	O1D—C21D—C22D—C23D	104 (2)
C21C—C22C—C23C—O3C	1 (4)	C24D—C21D—C22D—C23D	−16 (2)
C22C—C23C—O3C—C24C	−19 (3)	C21D—C22D—C23D—O3D	33 (2)
C23C—O3C—C24C—C21C	28 (5)	C22D—C23D—O3D—C24D	−37 (2)
O1A—C21C—C24C—O3C	−150 (4)	C23D—O3D—C24D—C21D	27 (3)
C22C—C21C—C24C—O3C	−26 (6)	O1D—C21D—C24D—O3D	−128 (2)
C8B—N1B—C1B—N2B	1.9 (7)	C22D—C21D—C24D—O3D	−5 (3)
C2B—N2B—C1B—N1B	0.7 (7)		

Symmetry codes: (i) −*x*+1, −*y*, *z*; (ii) −*y*+1/2, *x*−1/2, *z*; (iii) *y*+1/2, −*x*+1/2, *z*; (iv) −*x*+2, −*y*+1, *z*; (v) −*y*+3/2, *x*−1/2, *z*; (vi) *y*+1/2, −*x*+3/2, *z*.

### Hydrogen-bond geometry (Å, º)

**Table:**

*D*—H···*A*	*D*—H	H···*A*	*D*···*A*	*D*—H···*A*
N4*A*—H4*A*1···O4*B*^vii^	0.88	1.94	2.804 (5)	166
N3*A*—H3*A*···O2*A*^viii^	0.88	2.22	3.088 (5)	167
O4*A*—H4*C*···N5*A*	0.85 (5)	1.97 (5)	2.816 (6)	173 (4)
O4*A*—H4*D*···O5*A*	0.86 (5)	1.91 (5)	2.759 (6)	169 (5)
O5*A*—H5*C*···O2*A*	0.85 (3)	2.00 (4)	2.844 (4)	174 (5)
O5*A*—H5*D*···N1*B*^viii^	0.83 (5)	1.98 (5)	2.775 (6)	161 (5)
C4*A*—H4*A*···O2*A*^viii^	0.95	2.31	3.246 (6)	168
C16*A*—H16*A*···O4*B*^vii^	0.95	2.41	3.183 (6)	139
C22*A*—H22*A*···O3*A*^vi^	0.99	2.40	3.353 (13)	162
C24*A*—H24*A*···O5*B*^vii^	0.99	2.50	3.388 (14)	150
N4*B*—H4*B*1···O4*A*^ix^	0.88	1.96	2.820 (5)	167
N3*B*—H3*B*···O2*B*^x^	0.88	2.26	3.123 (5)	166
O4*B*—H4*E*···N5*B*	0.87 (5)	1.95 (5)	2.811 (5)	175 (6)
O4*B*—H4*F*···O5*B*	0.86 (4)	1.91 (4)	2.756 (5)	169 (4)
O5*B*—H5*E*···O2*B*	0.85 (4)	1.98 (3)	2.828 (4)	173 (5)
O5*B*—H5*F*···N1*A*^x^	0.86 (4)	1.95 (4)	2.794 (5)	169 (5)
C4*B*—H4*B*···O2*B*^x^	0.95	2.34	3.280 (6)	169
C16*B*—H16*B*···O4*A*^ix^	0.95	2.42	3.197 (6)	139
C22*B*—H22*D*···O5*A*^ix^	0.99	2.43	3.160 (8)	130
C24*B*—H24*D*···O3*B*^iii^	0.99	2.57	3.455 (9)	149

Symmetry codes: (iii) *y*+1/2, −*x*+1/2, *z*; (vi) *y*+1/2, −*x*+3/2, *z*; (vii) *x*+1/2, −*y*+1/2, −*z*−1; (viii) −*y*+1, −*x*+1, −*z*; (ix) *x*−1/2, −*y*+1/2, −*z*; (x) −*y*+1, −*x*+1, −*z*−1.
